# An integrated transcriptome and physiological analysis of nitrogen use efficiency in rice (*Oryza sativa L. ssp. indica*) under drought stress

**DOI:** 10.3389/fgene.2024.1483113

**Published:** 2024-11-01

**Authors:** Yu Wang, Yufan Zhang, Han Qiao, Yutong Zheng, Xin Hou, Liangsheng Shi

**Affiliations:** ^1^ State Key Laboratory of Water Resources Engineering and Management, Wuhan University, Wuhan, Hubei, China; ^2^ Center for Eco-Environmental Research, Nanjing Hydraulic Research Institute, Nanjing, Jiangsu, China; ^3^ State Key Laboratory of Hybrid Rice, Hubei Hongshan Laboratory, College of Life Sciences, Wuhan University, Wuhan, China

**Keywords:** pot experiment, nitrogen use efficiency, drought stress, transcriptome, physiology

## Abstract

Nitrogen is a critical nutrient vital for crop growth. However, our current understanding of nitrogen use efficiency (NUE) under drought remains inadequate. To delve into the molecular mechanisms underlying NUE under drought, a transcriptome and physiological co-expression analysis was performed in rice, which is particularly sensitive to drought. We conducted a pot experiment using rice grown under normal irrigation, mild drought stress, and severe drought stress. Compared to the normal treatment, drought stress led to a significant reduction in NUE across growth stages, with decreases ranging from 2.18% to 31.67%. Totals of 4,424 and 2,452 genes were identified as NUE-related DEGs that showed differential expressions (DEGs) and significantly correlated with NUE (NUE-related) under drought in the vegetative and reproductive stages, respectively. Interestingly, five genes involved in nitrogen metabolism were found in the overlapped genes of these two sets. Furthermore, the two sets of NUE-related DEGs were enriched in glyoxylate and dicarboxylate metabolism, as well as carbon fixation in photosynthetic organisms. Several genes in these two pathways were identified as hub genes in the two sets of NUE-related DEGs. This study offers new insights into the molecular mechanism of rice NUE under drought in agricultural practices and provides potential genes for breeding drought-resistant crops with high NUE.

## 1 Introduction

As a rain-fed crop, rice is particularly susceptible to various abiotic stresses, with drought stress being one of the most significant hindrances to its growth (Aslam et al., 2022; Mumtaz et al., 2020; Zhang et al., 2018). With climate change, the frequency and intensity of droughts are on the rise, exerting adverse effects on rice production (Giri et al., 2021; Korres et al., 2017; Pareek et al., 2020).

Nitrogen (N) is a vital nutrient crucial for plant growth and development. Paddy fields provide a growing environment for rice. The soil-water system in paddy fields can lead to N loss through processes such as ammonia volatilization, denitrification, and leaching, resulting in low N fertilizer use efficiency (Cao et al., 2016; Pang and Peng, 2010). When exploring the strategies to improve N use efficiency from the paddy field perspective, it is also essential to conduct relevant research on rice itself (Fan et al., 2008; Fan et al., 2011). The concept of nitrogen use efficiency (NUE) reflects the efficiency in converting input into output, with NUE for rice defined as the ratio of the shoot weight to unit N in shoots (Raghuram and Sharma, 2019). Drought stress significantly increases the N concentration in rice stems and leaves, leading to reduced N accumulation due to decreased dry matter accumulation (Niu et al., 2016; Zhu et al., 2017). Numerous studies have investigated the effects of drought stress on N metabolism, including its absorption, assimilation, and remobilization. Rice acquires N from the soil, with the inorganic N in the soil serving as the primary source. The available form of soil inorganic N dynamically changes with variations in soil water contents (Plett et al., 2020). NH_4_^+^ predominates in flooded soil, whereas NO_3_^−^ prevails in dried soil (Malyan et al., 2021). An experiment utilizing polyethylene glycol (PEG) to induce drought stress in hydroponics demonstrated that NO_3_^−^-treated rice exhibited lower N absorption compared to NH_4_^+^-treated rice (Cao et al., 2018). Various studies have examined the activities of crucial enzymes involved in N metabolism, including nitrate reductase (NR), nitrite reductase (NiR), glutamine synthetase (GS), glutamate synthase (GOGAT), and glutamate dehydrogenase (GDH) (Cao et al., 2018; Zhong et al., 2018; Sánchez-Rodríguez et al., 2011; Xu and Zhou, 2006). However, the findings have been inconsistent. NR and NiR activities were increased in rice roots but decreased in rice leaves under PEG-induced drought stress (Cao et al., 2018), while an increase in NR activity in rice leaves was observed in a pot experiment under drought (Zhong et al., 2018). Drought stress inhibited GS and GOGAT activities and enhanced GDH activity in tomatoes, with the exception of one stress-tolerant cultivar (Sánchez-Rodríguez et al., 2011). The inhibitory effects of drought stress on NR, GS, and GDH activities gradually intensified with the severity and duration of the stress (Xu and Zhou, 2006). In addition, GS and GOGAT are also crucial in the recycling of ammonium released during photorespiration, an energy dissipation side reaction of photosynthesis (Kendall et al., 1986; Somerville and Ogren, 1980; Zeng et al., 2017). Photosynthesis in plants plays a principal role in converting solar energy into biomass. Drought typically leads to a reduction in photosynthesis in C3 plants followed by reduced biomass accumulation. Photorespiration is beneficial in maintaining photosynthesis in C3 plants under drought by reducing photoinhibition and supplying ribulose-1,5-bisphosphate (RUBP) (Flexas and Medrano, 2002; Wingler et al., 2000). However, the enhanced photorespiration in rice under drought may lead to a higher net loss of carbon and N in the forms of CO_2_ and NH_3_ released primarily through the glycine decarboxylation in the mitochondria, one important step of photorespiration (Kaachra et al., 2018). Meanwhile, the photorespiratory pathway can fix carbon in the forms of amino acids, thus increasing the rate of photosynthetic CO_2_ uptake (Busch et al., 2018). Amino acids, important nitrogenous compounds, that accumulate in large quantities under drought, can help plants defend against drought by contributing to osmotic regulation (Ashraf and Foolad, 2007; Li et al., 2010). There is a complex interaction between carbon metabolism and N metabolism. Therefore, more investigations are necessary to elucidate NUE and the underlying mechanisms under drought stress in rice.

Crop traits are determined by the intricate interplay between the genotype (internal factor) and the environment (external factor). Drought stress can influence the expression of certain genes associated with N metabolism (N-responsive genes) and the overexpression of specific genes can regulate crop growth under drought stress. In a previous study, drought stress upregulated the expression of two *ammonium transporters* (*AMT1;2* and *AMT1;3*), while most *nitrate transporters* (*NARs* and *NRTs*) were downregulated (Cao et al., 2018). In flooded soil, the main inorganic N transporters in rice roots were *ammonium transporters* (*OsAMT1* and *OsAMT2* families) and *nitrate transporters* (*OsNPF2.4*, *OsNRT1.1A*, and *OsNRT2.3*), while, in upland soil, *nitrate transporters* (*OsNRT1.1B* and *OsNRT1.5A*) predominated (Wang et al., 2020). Furthermore, chloroplastic *GS2* (*OsGS2*) genes, along with one cytosolic *GS1* (*OsGS1;1*) gene, were implicated in regulating drought tolerance in a drought-tolerant cultivar (Singh and Ghosh, 2013). The overexpression of a cytosolic *GS1* (*GS1;2*) gene in rice exacerbated its drought sensitivity compared to the wild type (Cai et al., 2009). Research has indicated that *drought and salt tolerance* (*DST*), a zinc-finger transcription factor, can modulate the *NR 1.2* (*OsNR1.2*) gene, thus participating in nitrate assimilation under drought stress (Han et al., 2022). The co-overexpression of *OsNRT2.3a* and one *nitrate transporter partner protein 2* (*OsNAR2.1*) gene has been shown to enhance agronomic NUE (Chen et al., 2020). However, there has been limited focus on genes contributing to NUE. Some scholars have recognized this gap and identified NUE-related genes (Kumari et al., 2021; Sharma et al., 2023). In these studies, NUE-related genes were identified as those that were simultaneously N-responsive and yield-related, while NUE was defined as yield per unit of N (i.e., NUE_grain_, not NUE). Additionally, there may be shortcomings in the method used to identify NUE-related genes, as these studies primarily relied on the analysis of public data rather than direct NUE observations. It is crucial to define NUE-related genes as those directly involved in regulating NUE on the basis of real observations. Therefore, there is a need to comprehensively explore NUE-related genes at the whole-genome level based on actual NUE observations. Furthermore, research examining NUE-related genes under drought stress is notably lacking.

Transcriptomics represents the most promising avenue for unraveling the molecular intricacies of NUE. It allows for pinpointing differentially expressed genes (DEGs) under specific conditions, elucidating the physiological and phenotypic variations in crops on a genome-wide scale (Ahmad, 2022; Love et al., 2014). Several researchers have substantiated various functions, such as plant hormone signal transduction and carbon and N metabolism, implicated in the drought response at the molecular level (Ereful et al., 2020; Kaur et al., 2023; Park and Jeong, 2023; Zhang et al., 2017). These findings have furnished candidate genes for the development of drought-tolerant rice. Some candidate genes, like *osmotic stress/ABA–activated protein kinase 3* (*OsSAPK3*), *OsNAR2.1*, and *NAM/ATAF/CUC (NAC) domain transcription factor5* (*OsNAC5*), have been confirmed to enhance drought tolerance in rice through over-expression experiments (Chen et al., 2019; Jeong et al., 2013; Lou et al., 2023; Lv et al., 2023). However, these investigations have predominantly focused on DEGs and their molecular functions under drought stress and have not explored the correlation between these DEGs and external traits (phenotypic and physiological traits) under drought stress. A classical method for identifying genes that regulate external traits is weighted gene co-expression network analysis (WGCNA) (Langfelder and Horvath, 2008). Based on systems biology, this approach can categorize highly coordinated gene sets, known as modules. By examining the relationship between gene networks within these modules and external traits, WGCNA helps pinpoint hub modules and hub genes that regulate these traits. Several studies have utilized WGCNA to identify hub genes or molecular mechanisms governing various external traits, such as biomass (Xu et al., 2023), ear leaf N concentration (Gong et al., 2020a), and organic acid content (Wang et al., 2022a), among others, in response to growth disparities (Gong et al., 2020a) or environmental fluctuations (Wang et al., 2022b; Xu et al., 2023). The research of a previous study treated NUE_grain_ as an external trait and identified two network modules highly correlated with NUE_grain_ (Shanks et al., 2022). However, research on the highly correlated modules of NUE and NUE-related genes under drought stress is still lacking. In this study, we first applied WGCNA to identify NUE-related genes using data from a pot experiment. Subsequently, we identified DEGs associated with NUE under drought stress from the pool of NUE-related genes. This enabled us to uncover the hub genes among NUE-related DEGs that regulate NUE under drought stress.

The aim of this study is to enhance our understanding of the physiological changes and molecular mechanisms in rice NUE under drought stress. To achieve this, we conducted a pot experiment involving three different treatments: normal irrigation, mild drought stress, and severe drought stress. Throughout the experiment, we closely monitored the growth of the rice plants and analyzed the transcriptome of their leaves. Through the integration of rice transcriptome and physiological data, we identified two sets of candidate genes, referred to as NUE-related DEGs, which are likely to play pivotal roles in rice NUE under drought stress. Furthermore, we conducted functional analyses of these two sets of NUE-related DEGs and identified transcription factors, transporters, and protein kinases among them. Overall, this study is expected to shed light on the potential regulatory mechanisms involved in NUE adaptation to water-limited environments and provide a valuable gene list for developing drought-resistant crops with high NUE.

## 2 Materials and methods

### 2.1 Plant materials, growth conditions, and stress treatments

In this study, we utilized the indica rice variety “Zuan Liang You Chao Zhan”. Thirty days after sowing, on 31 May 2021, the seedlings were transplanted into experimental pots at the Irrigation and Drainage Experimental Site of the State Key Laboratory of Water Resources Engineering and Management, Wuhan University (114°37′E, 30°54′N). The harvest took place on 12 October 2021. The experimental pots were filled with 6.4 kg of air-dried soil. Based on the USDA soil texture classification, the soil used in this study was identified as silt loam with 70.75% silt, 14.09% sand, and 15.15% clay. The total N content of the soil was 0.67 g kg^−1^.

We implemented three treatments: normal irrigation (WF), mild drought (WM), and severe drought (WS). In the WF treatment, a 5–50 mm water layer was maintained throughout the entire growth period. The WM and WS treatments had a 5–50 mm water layer until the tillering stage, after which the soil water potentials were maintained as the designed soil water potentials (−15 ± 5 kPa and −30 ± 5 kPa for WM and WS treatments, respectively). We monitored soil water contents to control the soil water potentials and the corresponding soil water contents at the designated soil water potentials were obtained by a soil water retention curve (Supplementary Figure S1A). To prevent the influence of rainfall, the rain shelter was closed on rainy days during the experiment. Then the designed soil water potentials were maintained through artificial water replenishment. Significant differences in soil moisture were observed across all four growth stages (Supplementary Figure S1B). Throughout the experiment, all treatments (WF, WM, and WS treatments) received the same adequate amount of nitrogen fertilizer. Additionally, each treatment included three pots, designated as WF-0, WM-0, and WS-0 treatments, without fertilizer during the experiment. The pots in the WF-0, WM-0, and WS-0 treatments were subjected to the same water management as their corresponding treated pots (WF, WM, and WS treatments).

### 2.2 Soil properties

Soil samples from the pots in the WF, WM, and WS treatments were collected at the four main growth stages, with three repetitions per treatment. To ensure the comparability of soil samples across treatments, each soil sample was taken from the exact center of the pot after the pot was destructed. Each soil sample was divided into two portions: one for measuring soil moisture using the drying method, and the other for determining soil inorganic N concentrations utilizing an ultraviolet and visible light spectrophotometer (UV2700, Shimadzu, Japan). The soil inorganic N concentrations include soil NO_3_^−^-N and NH_4_^+^-N concentrations.

### 2.3 Rice physiological and morphological traits

Following the soil sample collection process described above, the aboveground parts of rice plants grown under WF, WM, and WS treatments were sampled with three biological replicates per treatment. The aboveground rice samples in each treatment were taken from the same three pots as the three soil samples. The aboveground rice was further divided into leaf, stem, and panicle components. The fresh weight (FW) of each tissue was recorded immediately after it was isolated from the aboveground rice. Subsequently, each plant tissue was dried at 105 °C for 30 min to deactivate enzymes and then at 80 °C until a constant weight was achieved, from which the dry weight (DW) was determined. The dried samples were then powdered, and the N concentration in each tissue was determined using the Kjeldahl method (Nelson and Sommers, 1973). Tissue N accumulation was calculated by multiplying the tissue DW by the tissue N concentration. The total aboveground N accumulation was obtained by summing up the N accumulation results for each aboveground tissue. The aboveground N concentration was determined as the N accumulation per unit of DW of the aboveground rice. The NUE can be calculated using Equation 1, as described in a previous study (Somaweera et al., 2016):

$$\text{NUE}=\frac{\text{DW}}{N}$$ (1)

where NUE (g·g^−1^) is the nitrogen use efficiency, DW (g) is the aboveground dry weight, and N (g) is the aboveground N accumulation.

During the harvest period, aboveground rice in the WF, WM, WS, WF-0, WM-0, and WS-0 treatments was collected and divided into leaf, stem, and panicle components. The rice yield was determined based on the actual weight of the harvested grain in the pot. The aboveground DW, N accumulation, and NUE were determined using the same methods as described earlier.

### 2.4 Transcriptome analysis method

Leaf samples were collected concurrently with soil samples, with three biological replicates per treatment. Leaves were promptly excised from the plants, flash-frozen in liquid nitrogen, and transported to the Majorbio laboratory (Shanghai, China) for cryopreservation at −80°C. Transcriptome-wide analysis was conducted using RNA sequencing (RNA-seq). Total RNA was extracted, purified, and fragmented, followed by the construction of cDNA libraries. Subsequently, high-throughput sequencing was performed, and the resulting clean reads were aligned to the rice genome. Normalization of the data was carried out as transcripts per kilobase of exon model per million mapped reads (TPM) using RNA-Seq by Expectation Maximization (RSEM) (version 1.3.3) (Li and Dewey, 2011). The analyses were conducted on the online Majorbio Cloud Platform (www.majorbio.com). To identify DEGs, we employed DESeq2 (Version 1.24.0) with replicates, setting a false discovery rate (FDR) of <0.05, a fold change (FC) value of ≥2, and an adjusted *p*-value (*p*-adjust) of <0.05 as thresholds. Raw sequence data were submitted to the National Center for Biotechnology Information (NCBI) under the BioProject Submission ID PRJNA1108788 and used for bioinformatic analyses.

### 2.5 Gene co-expression network analysis and NUE-related gene

WGCNA (Version 1.63) was employed for gene co-expression network analysis, and conducted separately for the vegetative and reproductive stages. First, genes with TPM values <1 and coefficients of variation <0.1 were filtered out. Subsequently, the soft-thresholding power (β) was determined based on the principle of scale-free topology. Module identification was then performed by setting the β value, the minimum number of genes per module (30), and the minimum eigengene-based module membership (0.30). Additionally, modules with a cluster distance below 0.25 were merged. Genes exhibiting similar expression patterns were grouped into the same module, while genes with dissimilar expression patterns were classified into the grey module. To conduct the analysis, we selected aboveground dry weight (ADW), aboveground nitrogen concentration (AN), aboveground nitrogen accumulation (ANA), and NUE in rice as the traits of interest. Pearson correlation was utilized to identify the hub modules. The genes belonging to these hub modules were aggregated to form the NUE-related gene set for subsequent analysis. Gene interactions were visualized using Cytoscape (version 3.6.0). The gene interactions were derived from the corresponding protein-protein interactions (PPIs) available in the STRING database version 11 (https://cn.string-db.org).

### 2.6 Functional enrichment analysis

Gene Ontology (GO) enrichment analysis and Kyoto Encyclopedia of Genes and Genomes (KEGG) enrichment analysis were conducted using Goatools (https://github.com/tanghaibao/GOatools) (Klopfenstein et al., 2018) and KOBAS (http://kobas.cbi.pku.edu.cn/home.do) (Xie et al., 2011), respectively. A GO term or KEGG pathway was considered enriched when the *p*-value was <0.05 and statistically significant when the Bonferroni-corrected *p*-value (*p*-adjust) was lower than 0.05.

### 2.7 Data mining for NUE-related DEGs

We focused on transcription factors (TFs), transporters, and protein kinases (PKs), which are known to play key roles in abiotic stress signaling and responses in plants (Gong et al., 2020b; Zhu, 2016). TFs were classified using the PlantTFDB database (http://planttfdb.gao-lab.org) (Jin et al., 2017), transporters were identified based on the Rice Transporters Database (https://ricephylogenomics.ucdavis.edu/transporter/), and PKs were recognized using the iTak database (http://itak.feilab.net/cgi-bin/itak/index.cgi) (Zheng et al., 2016).

### 2.8 Verification of RNA-seq

To validate the transcriptome sequencing results, real-time quantitative reverse transcription polymerase chain reaction (qRT-PCR) was conducted on five randomly selected DEGs. The primer sequences for these genes are provided in Supplementary Table S2. Each sample underwent three biological replications, and eEF1-α was used as the reference gene. The relative expression of DEGs was calculated using the 2^−ΔΔCT^ method (Livak and Schmittgen, 2001).

### 2.9 Statistical analysis

Statistical analyses were performed using SPSS statistical software (version 11.5, SPSS Inc., Chicago, IL, USA, 2003). Repeated measures analysis of variance (ANOVA) was employed to test for differences among treatments at a significance level of 0.05. Least significant difference (LSD) tests were used to compare the means of the soil inorganic N and rice traits among the WF, WM, and WS treatments in the four main growth stages. Duncan’s multiple-comparison tests were performed to determine the differences in rice traits among all treatments during the harvest period. The results of soil properties and rice traits were visualized using Origin 2021b.

## 3 Results

### 3.1 Soil inorganic N

**FIGURE 1:**
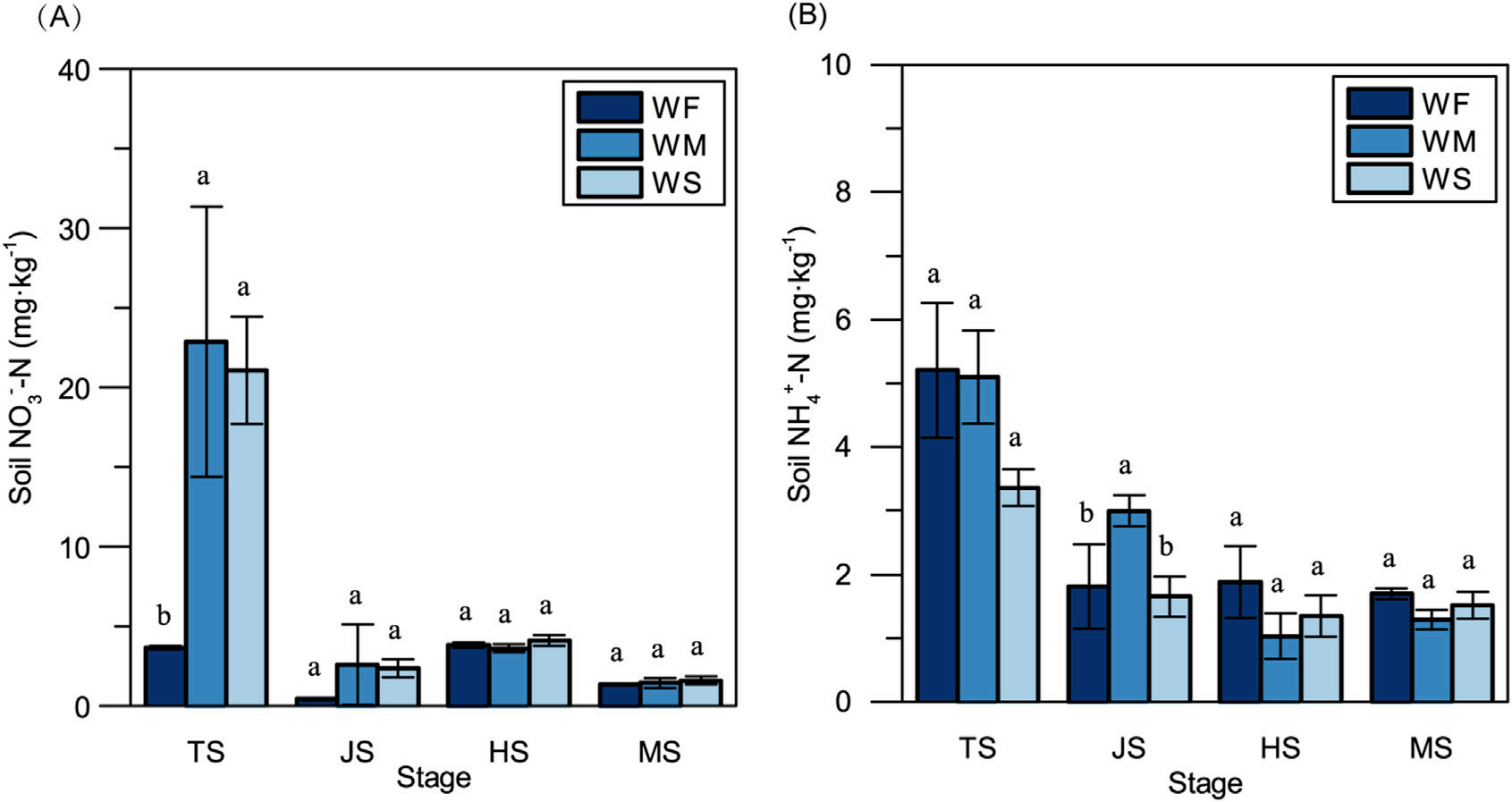
The soil NO_3_^−^-N concentration **(A)** and NH_4_^+^-N concentration **(B)** across three treatments at the four main growth stages. WF, WM, and WS denote normal irrigation, mild drought stress, and severe drought stress, respectively, while TS, JS, HS, and MS represent the tillering, jointing, heading, and maturing stages. The data and error bars in the figure represent the average value and standard deviation of three repetitions, respectively. Different lowercase letters for the same growth stage denote significant differences at 0.05 level by LSD test.

Fluctuations in soil moisture can have a notable impact on soil inorganic N concentrations. In the tillering stage, soil NO_3_^−^-N concentrations in the WM and WS treatments were significantly elevated compared to the WF treatment, exhibiting increases of 525.94- and 477.07-folds, respectively (Figure 1A). Furthermore, because fertilizer was applied additionally during the early heading stage, NO_3_^−^-N concentrations in the heading stage surpassed those observed in the jointing stage in all treatments. NH_4_^+^-N concentrations in all three treatments were notably higher at the tillering stage compared to other stages (Figure 1B). Notably, significant disparities in soil NH_4_^+^-N concentrations among the three treatments only emerged in the jointing stage.

### 3.2 Rice physiological and morphological traits

**FIGURE 2:**
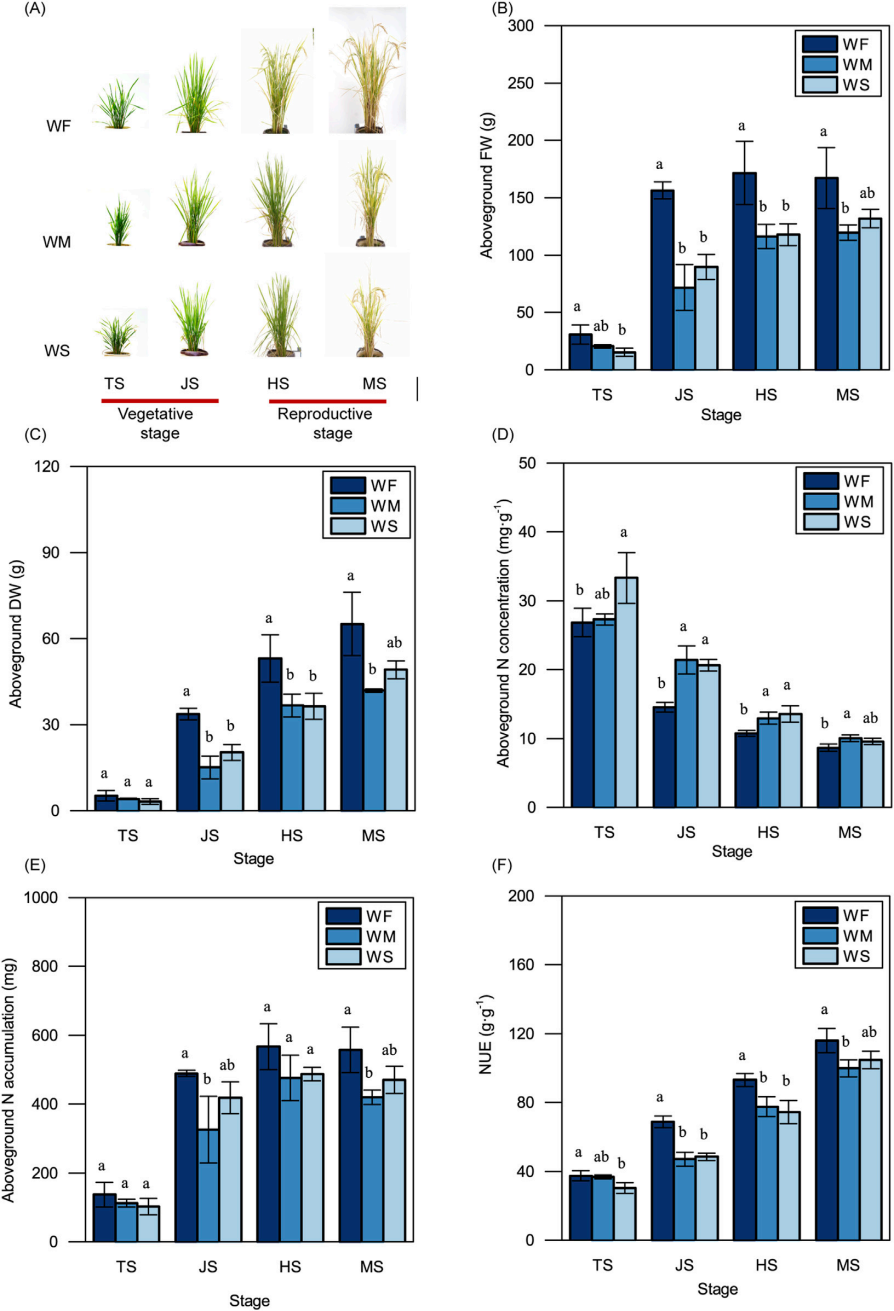
Rice plant growth. **(A)** Rice phenotype. Bar = 20 cm. **(B–F)** The variations in the aboveground fresh weight (FW) **(B)**, dry weight (DW) **(C)**, nitrogen concentration (N concentration) **(D)**, nitrogen accumulation (N accumulation) **(E)**, and nitrogen use efficiency (NUE) **(F)**. WF, WM, and WS denote normal irrigation, mild drought stress, and severe drought stress, respectively, while TS, JS, HS, and MS represent the tillering, jointing, heading, and maturing stages. The data and error bars in the figure represent the average value and standard deviation of three repetitions, respectively. Different lowercase letters for the same growth stage denote significant differences at the 0.05 level according to the LSD test.

As shown in Figure 2A, rice plants subjected to WM and WS treatments were smaller and more compact in both the vegetative and reproductive stages compared to those in the WF treatment. Plants in the WF treatment consistently displayed significantly higher aboveground FWs compared to those in the WM and WS treatments across all four growth stages (Figure 2B). Notably, aboveground FWs in all three treatments experienced rapid increases during the vegetative stage, particularly in the WF treatment. Subsequently, in the heading stage, the aboveground FWs in all three treatments slightly increased, with no significant changes observed in the maturing stage. The trends in aboveground DWs among the WF, WM, and WS treatments across all four growth stages mirrored those in aboveground FWs (Figure 2C). Across all growth stages, the WF treatment consistently displayed significantly lower aboveground N concentrations and higher aboveground N accumulations compared to the WM and WS treatment (Figures 2D, E). Furthermore, rice in the WF treatment consistently exhibited higher NUE compared to rice in the WM and WS treatments at all growth stages, with differences ranging from 2.23% to 46.36% (Figure 2F). These results showed drought stress led to a significant reduction in NUE across growth stages, compared to the normal treatment.

The pots without fertilizer (i.e., WF-0, WM-0, and WS-0) exhibited significantly lower grain yields, panicle numbers, and aboveground DWs compared to fertilized pots (i.e., WF, WM, and WS) within each treatment (Table 1). Despite higher aboveground N accumulation in the fertilized treatments compared to the treatments without fertilizer, the NUE in the fertilized treatments decreased with a significant difference. Among the fertilized treatments, the highest grain yield was observed in the WF treatment, reaching 28.54 g·pot^−1^. Specifically, the aboveground DW in the WF treatment was significantly higher than those in the WM and WS treatments. However, the aboveground N accumulation and NUE did not significantly differ among the WF, WM, and WS treatments.

**TABLE 1:** The effects of different treatments on the grain yield and NUE.

Treatment	Grain yield (g·pot^−1^)	No. Panicle (pot^−1^)	Spikelets (panicle^−1^)	Grain weight (g·pot^−1^)	Aboveground DW (g·pot^−1^)	Aboveground N accumulation (mg·pot^−1^)	NUE (g·g^−1^)
WF	(28.54 ± 6.16)a	(14.00 ± 0.82)a	(92.29 ± 9.92)a	(19.60 ± 0.62)ab	(63.60 ± 7.96)a	(600.52 ± 140.31)a	(108.39 ± 10.84)b
WM	(21.29 ± 1.49)b	(13.00 ± 0.00)a	(83.24 ± 4.23)ab	(18.13 ± 0.37)b	(52.77 ± 4.08)b	(478.02 ± 23.89)a	(110.34 ± 5.27)b
WS	(21.71 ± 1.95)b	(13.67 ± 0.47)a	(73.57 ± 7.46)b	(18.38 ± 0.93)ab	(51.21 ± 3.74)b	(517.89 ± 35.87)a	(99.10 ± 7.25)b
WF-0	(8.31 ± 1.99)c	(5.50 ± 0.41)d	(81.37 ± 10.59)ab	(18.08 ± 0.75)b	(29.74 ± 2.20)c	(208.86 ± 16.04)b	(142.42 ± 0.39)a
WM-0	(12.89 ± 1.56)c	(7.33 ± 0.94)c	(88.35 ± 3.13)ab	(19.95 ± 0.83)a	(31.72 ± 2.45)c	(244.50 ± 16.07)b	(129.64 ± 2.29)a
WS-0	(12.70 ± 0.98)c	(9.00 ± 0.82)b	(74.49 ± 6.80)ab	(19.06 ± 0.57)ab	(29.69 ± 1.46)c	(216.64 ± 21.48)b	(137.94 ± 9.79)a

Nitrogen use efficiency (NUE) values for normal irrigation (WF), mild drought stress (WM), and severe drought stress (WS) treatments are provided in the table above. Different lowercase letters in each column denote significant differences at the 0.05 level according to Duncan’s multiple comparison test. The values represent the mean value ±standard error (n = 3).

### 3.3 Transcriptome results and validation

To delve into the molecular intricacies of NUE regulation during drought stress in rice, we employed RNA-seq to track gene expression profiles. Transcriptome sequencing was conducted on rice leaves in the WF, WM, and WS treatments at the tillering (1), jointing (2), heading (3), and maturing (4) stages (Supplementary Table S2). The lowest values of Q20, Q30, and the total mapping ratio were 97.66%, 93.39%, and 93.03%, respectively. Thus, all 36 leaf samples were suitable for subsequent analysis.

To validate the reliability of the RNA-seq data, we measured multiple genes using qRT-PCR. The Pearson correlation coefficient demonstrated a significant correlation between the log_2_FC values obtained from the RNA-seq and qRT-PCR results (Supplementary Figure S2). This result confirmed the reliability of RNA-seq.

### 3.4 DEG analysis under drought stress

**FIGURE 3:**
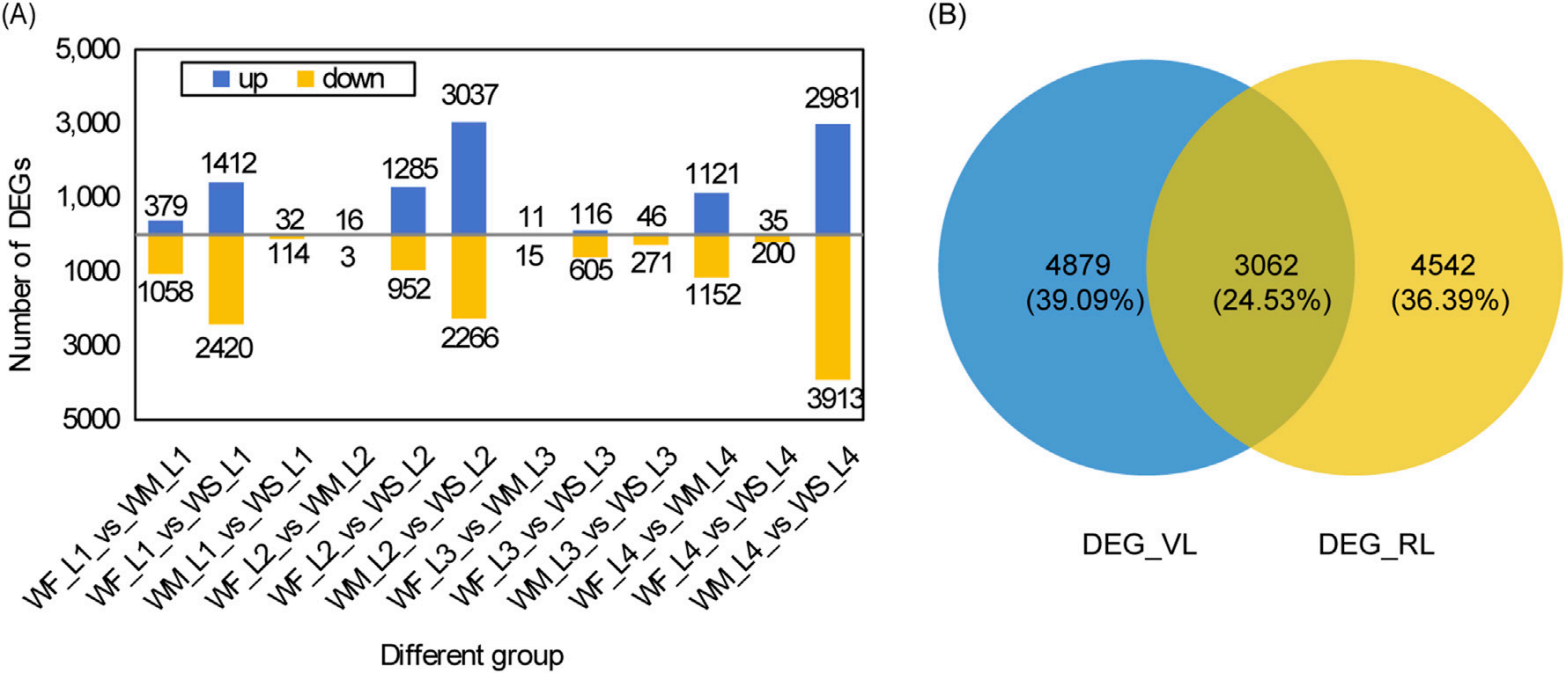
DEG analysis under drought stress. **(A)** Statistical analysis of DEGs across three treatments at four main growth stages. The three treatment were normal irrigation (WF), mild drought stress (WM), and severe drought stress (WS), and the four main growth stages were the tillering, jointing, heading, and maturing stages (represented by 1, 2, 3, and 4 respectively). The comparisons WF_vs._WM and WF_vs._WS represent differentially expressed gene (DEG) sets obtained by comparing WM and WS samples with the control WF samples, respectively. Similarly, WM_vs._WS denotes the DEG sets obtained by comparing WS samples with the control WM samples. **(B)** A Venn diagram of DEG_VL and DEG_RL. DEG_VL and DEG_RL denote DEGs at the vegetative and reproductive stages, respectively.

The rice leaf DEGs responsive to drought stress across various growth stages are depicted in Figure 3. The highest numbers of DEGs identified when comparing WM samples with the control WF samples (WF_vs._WM) appeared in the tillering and maturing stages, with 1,437 and 2,273 DEGs, respectively (Figure 3A). Similarly, comparing WS samples with the control WF samples (WF_vs._WS) showed the highest numbers of DEGs in the tillering and jointing stages, with 3,832 and 2,273 DEGs, respectively. The numbers of DEGs in WF_vs._WS were 2.67, 117.74, and 27.73 times higher than those in WF_vs._WM at the tillering, jointing, and heading stages, respectively. The highest numbers of DEGs obtained by comparing WS samples with the control WM samples (WM_vs._WS) were observed in the jointing and maturing stages, with 5,303 and 6,894 DEGs, respectively. Among the 12 different groups, except for the 3 groups in the jointing stage, the number of downregulated DEGs was greater than that of the upregulated DEGs.

We identified a total of 19, 0, 0, and 9 common DEGs among the three different groups (WF_vs._WM, WF_vs._WS, and WM_vs._WS) at the tillering, jointing, heading, and maturing stages, respectively (Supplementary Figure S3A). The DEG database for the tillering stage is denoted as DEG_L1 and was obtained by merging the DEGs from WF1_vs._WM1, WF1_vs._WS1, and WM1_vs._WS1. Similarly, the databases for the jointing, heading, and maturing stages are named DEG_L2, DEG_L3, and DEG_L4, respectively, and were created by following the same merging procedure. Only 36 DEGs, accounting for 0.29% of all DEGs, were shared among the four DEG databases (Supplementary Figure S3B). For the vegetative stage, merging DEG_L1 and DEG_L2 yielded 7,941 DEGs (DEG_VL) while for the reproductive stage, merging DEG_L3 and DEG_L4 produced 7,604 DEGs (DEG_RL) (Figure 3B). These two databases shared 3,062 DEGs, representing 24.53% of all DEGs.

### 3.5 Co-expression network reveals NUE-related genes

**FIGURE 4:**
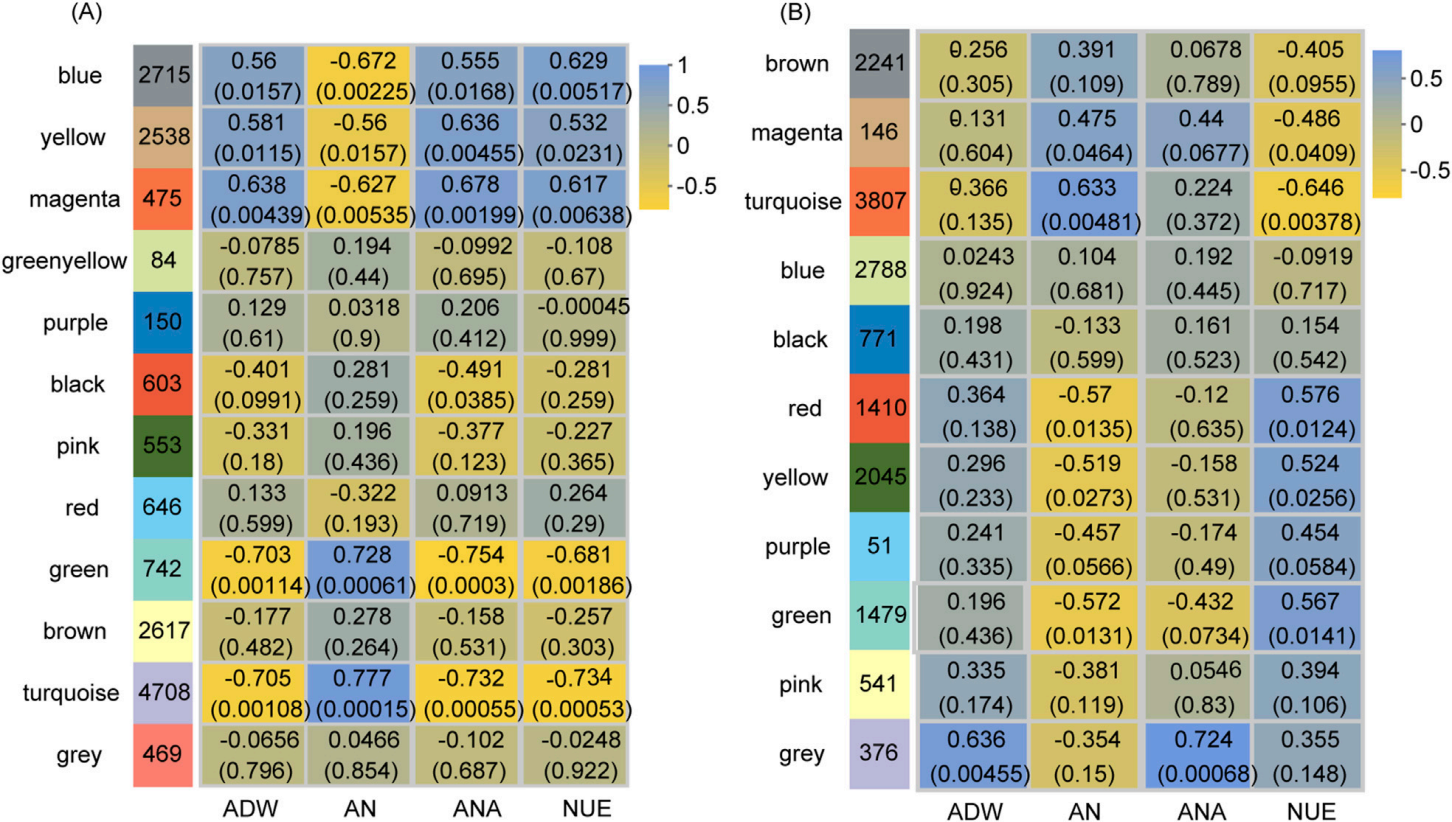
Module-trait relationships at the vegetative stage **(A)** and reproductive stage **(B)**. The *y*-axis represents the module names, while the *x*-axis denotes traits. ADW, AN, ANA, and NUE correspond to aboveground dry weight, aboveground nitrogen concentration, aboveground nitrogen accumulation, and nitrogen use efficiency, respectively. The numbers in the left column indicate the number of genes in each module, while each set of data on the right presents the correlation coefficient and significance *p*-value (in parentheses) of the module with the respective trait. The color legend illustrates the correlation level.

Utilizing WGCNA, we identified NUE-related gene sets in the vegetative and reproductive stages. After gene filtering, 16,300 genes in the vegetative stage and 15,655 genes in the reproductive stages were retained for analysis. The β value was determined to be 9 for both stages. Gene dendrograms were constructed based on gene expression patterns (Supplementary Figure S4A; Supplementary Figure S4B). According to these dendrograms, the filtered genes in the vegetative and reproductive stages were organized into 12 and 11 modules, respectively (Figure 4).

At the vegetative stage, the gene expression in five modules represented significant correlations (*p*-value <0.05) with NUE by the Pearson correlation analysis (Figure 4A). The expression of genes within the blue, yellow, and magenta modules had notable positive correlations with NUE, whereas that in the green and turquoise modules demonstrated significant negative correlations. The expression of genes within these five modules was also significantly correlated with ADW, AN, and ANA. Notably, the regulatory patterns observed in ADW and ANA mirrored those found in NUE, whereas the regulatory pattern in AN was opposite to that observed in NUE. Consequently, the genes in these five modules for the vegetative stage were combined and designated as NUE_VL, comprising 11,178 genes (Supplementary Table S3).

At the reproductive stage, the gene expression in the five modules had significant correlations (*p*-value <0.05) with NUE by the Pearson correlation analysis (Figure 4B). The expression of genes within the red, yellow, and green modules showed significant positive correlations with NUE, while that in the magenta and turquoise modules displayed significant negative correlations. Additionally, the expression of genes within these five modules was significantly correlated with AN, exhibiting the opposite regulation pattern. Therefore, the genes in these five modules for the reproductive stage were merged and named NUE_RL, consisting of 8,887 genes (Supplementary Table S3).

### 3.6 Functional analysis of NUE-related DEGs

**FIGURE 5:**
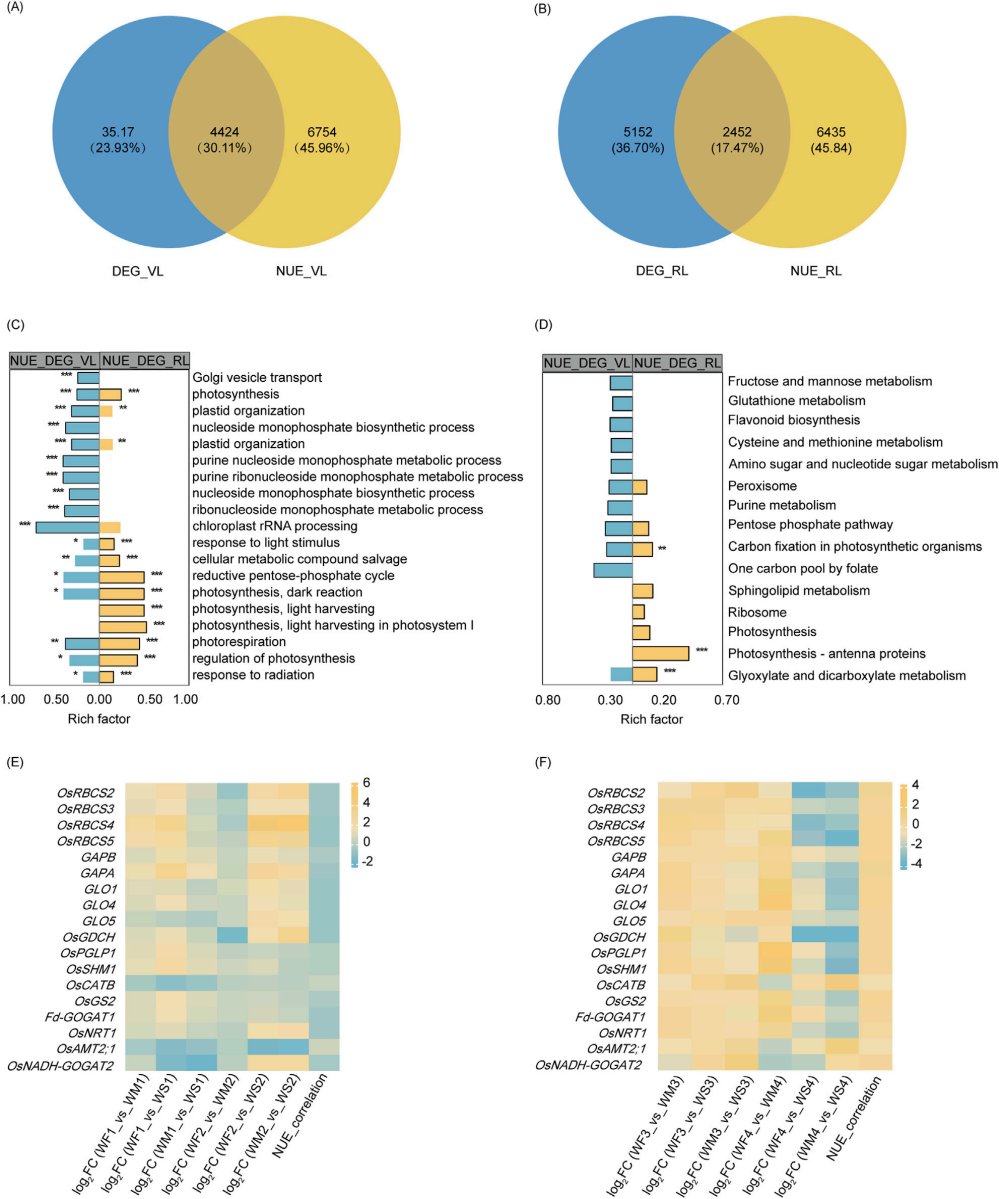
NUE-related DEG analysis. **(A, B)** A Venn diagram illustrating the overlap between drought-responsive DEGs and NUE-related genes in the vegetative stage **(A)** and reproductive stage **(B)**. DEG_VR and DEG_RR represent the differentially expressed genes (DEGs) in the vegetative and reproductive stages, respectively, while TN_VR and TN_RR denote the genes that show significant correlations with nitrogen use efficiency (NUE-related) in the vegetative and reproductive stages. **(C, D)** A display of the top 10 enriched (*p*-value <0.05) GO terms **(C)** and KEGG pathways **(D)** of two NUE-related DEG sets. DEG_TN_VR and DEG_TN_RR refer to the NUE-related DEGs in the vegetative and reproductive stages, respectively. Significance levels are indicated by * (*p*-adjust <0.05), ** (*p*-adjust <0.01), and *** (*p*-adjust <0.001). **(E, F)** A representation of the log2 fold change (log_2_FC) and nitrogen use efficiency (NUE) correlation coefficient of selected genes involved in glyoxylate and dicarboxylate metabolism, carbon fixation in photosynthetic organisms, and nitrogen metabolism across the three different groups IN the vegetative stage **(E)** and reproductive stage **(F)**.

To identify the DEGs among NUE-related genes under drought, Venn diagrams were generated for the vegetative stage (Figure 5A) and reproductive stage (Figure 5B). DEG_VL and NUE_VL shared 4,424 genes, forming a set denoted by DEG_NUE_VL. Similarly, DEG_RL and NUE_RL shared 2,452 genes, collectively designated as DEG_NUE_RL. In DEG_NUE_VL and DEG_NUE_RL, five genes were already known as the genes involved in N metabolism (Cai et al., 2010; Chen et al., 2016; Lin et al., 2000; Suenaga et al., 2003; Yamaya and Kusano, 2014). The five genes were the *nitrate transporter* (*OsNRT1*), the *MEP/AMT2-type ammonium transporter* (*OsAMT2.1*), the *OsGS2*, the *ferredoxin-dependent GOGAT 1* (*Fd-GOGAT1*), and the *NADH-GOGAT 2* (*OsNADH-GOGAT2*). These genes were found to be NUE-related DEGs in both stages. The expressions of *OsNRT1*, *OsGS2*, *Fd-GOGAT1*, and *OsNADH-GOGAT2* displayed significant negative and positive correlations with NUE in the vegetative and reproductive stages, respectively (Figures 5E, F). The correlation between the expressions of *OsAMT2.1* and NUE exhibited the opposite trend. These genes involved in N metabolism are likely to be crucial in rice NUE under drought.

GO enrichment analysis revealed that DEG_NUE_VL and DEG_NUE_RL shared 32 significantly enriched GO terms (*p*-adjust <0.05), including processes such as photosynthesis, oxidation-reduction process, biosynthetic process, and others (Supplementary Table S4; Figure 5C). KEGG enrichment analysis found that four enriched KEGG pathways were shared by both DEG_NUE_VL and DEG_NUE_RL (Supplementary Table S4; Figure 5D). Carbon fixation in photosynthetic organisms and glyoxylate and dicarboxylate metabolism were enriched in both gene lists and showed significant enrichment in DEG_NUE_RL.

The Calvin–Benson cycle and photorespiration are key mechanisms of carbon fixation in photosynthetic organisms and glyoxylate and dicarboxylate metabolism, respectively. In the vegetative stage, the expression of most genes involved in these processes was enhanced in the WM and WS treatments (Figure 5E). Genes with this enhanced expression included four *ribulose 1,5-bisphosphate carboxylase–oxygenase* (*Rubisco*) *small subunit* (*OsRBCS2-5*) genes, two *glyceraldehyde-3-phosphate dehydrogenase* (*GAPA* and *GAPB*) genes, the photorespiratory *2-phosphoglycolate phosphatase* (*OsPGLP1*) gene, three *glycolate oxidase* (*GLO1*, *GLO4*, and *GLO5*) genes, the *glycine decarboxylase complex H-protein* (*OsGDCH*) gene, the *serine hydroxymethyltransferase 1* (*OsSHM1*) gene, *OsGS2*, and *Fd-GOGAT1*. However, the expression of these genes was inhibited in the reproductive stage (Figure 5F). As NUE-related genes, their expression was negatively correlated with NUE in the vegetative stage, but positively correlated in the reproductive stage. However, the expression of the c*atalase isozyme B* (*OsCATB*) gene in the WM and WS treatments was inhibited in the vegetative stage but enhanced in the reproductive stage. Interestingly, the correlations between the expression of *OsCATB* and NUE were opposite to those observed with the above genes in both stages. The two pathways, carbon fixation in photosynthetic organisms and glyoxylate and dicarboxylate metabolism may play important roles in rice NUE under drought.

Based on a Venn analysis, the gene counts revealed that 43 genes of DEG_NUE_VL and 30 genes of DEG_NUE_RL were identified as NUE_grain_-related genes in previous studies (Kumari et al., 2021; Sharma et al., 2023) (Supplementary Table S4).

### 3.7 PPI network of NUE-related DEGs

**FIGURE 6:**
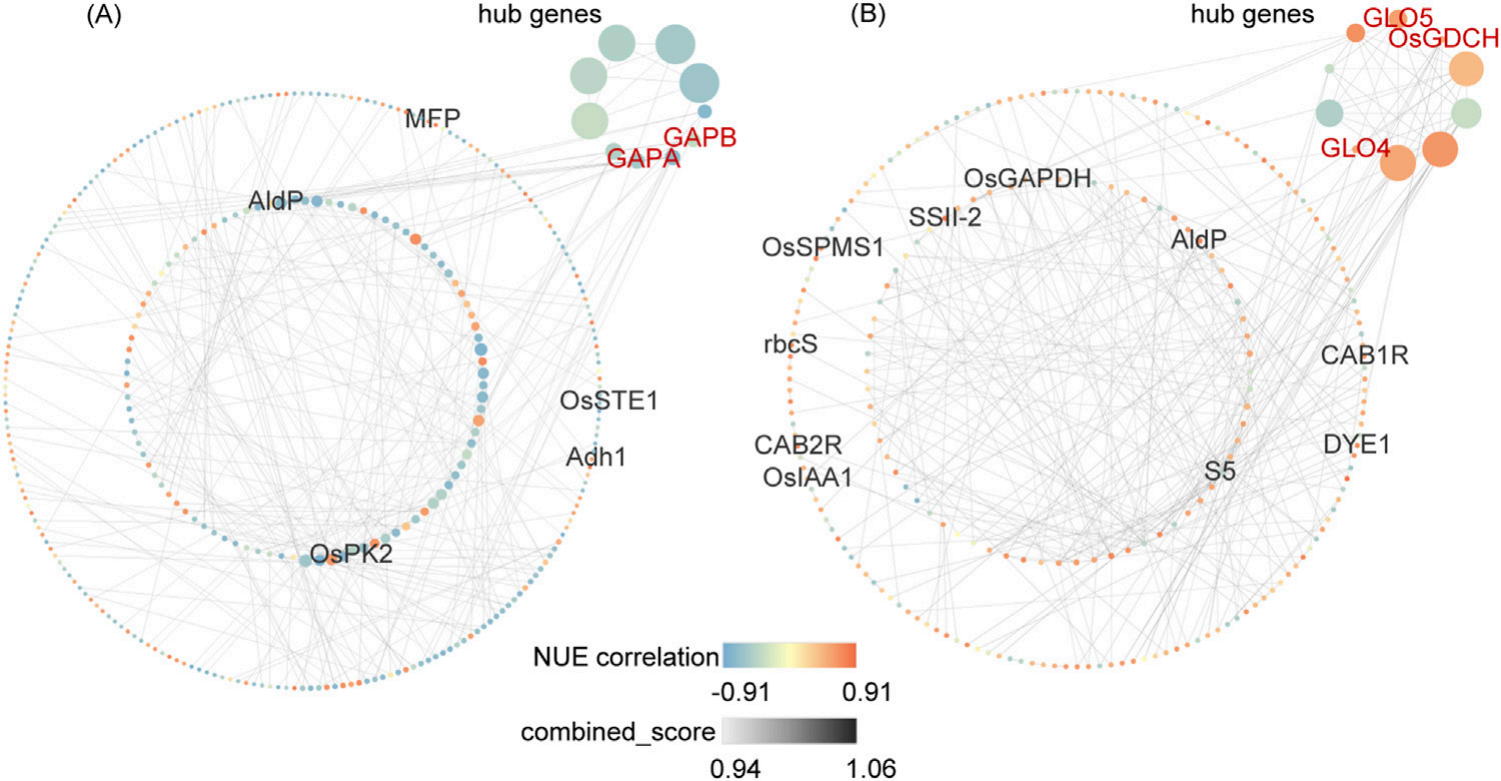
Top 300 interactions in protein-protein interaction (PPI) networks of DEG_NUE_VL **(A)** and DEG_NUE_RL **(B)**. DEG_NUE_VL and DEG_NUE_RL represent the differentially expressed genes (DEGs) that show significant correlations with nitrogen use efficiency (NUE-related) in the vegetative and reproductive stages, respectively. The node size represents the Maximal Clique Centrality (MCC) algorithm score. Black-labeled nodes indicate NUE_grain_-related genes, and red-labeled nodes represent genes involved in carbon fixation or glyoxylate and dicarboxylate metabolism.

To visualize the co-expression network of NUE-related DEGs, genes from DEG_NUE_VL and DEG_NUE_RL were selected for PPI network analysis. The interaction relationships were determined using combined scores obtained from the STRING database (Supplementary Table S5). Subsequently, we visualized NUE-related DEGs with the top 300 combined scores above 0.4 using Cytoscape (Figure 6). Specifically, 334 genes from DEG_NUE_VL are included in Figure 6A, while 238 genes from DEG_NUE_RL are represented in Figure 6B. To identify crucial proteins within the PPI network, we employed the Maximal Clique Centrality (MCC) algorithm available in the CytoHubba plugin of Cytoscape, a topology analysis method capable of accurately capturing essential proteins (Chin et al., 2014). Consequently, gene scores in the PPI network were calculated using the MCC algorithm, and the top 10 scoring genes were designated as hub genes (Figure 6). Notably, some genes involved in carbon fixation in photosynthetic organisms and glyoxylate and dicarboxylate metabolism emerged as hub genes. *GAPA* and *GAPB* were identified as two hub genes in the vegetative stage, while *GLO4*, *GLO5*, and *OsGDCH* were highlighted as three hub genes in the reproductive stage. These results indicated these several genes in the two significant enriched pathways may have major functions in rice NUE under drought.

Among the 300 interactions for DEG_NUE_VL, five NUE_grain_-related genes identified in previous studies (Kumari et al., 2021; Sharma et al., 2023) were included. Similarly, for DEG_NUE_RL, the top 300 interactions comprised 10 genes.

### 3.8 TFs in NUE-related DEGs

**FIGURE 7:**
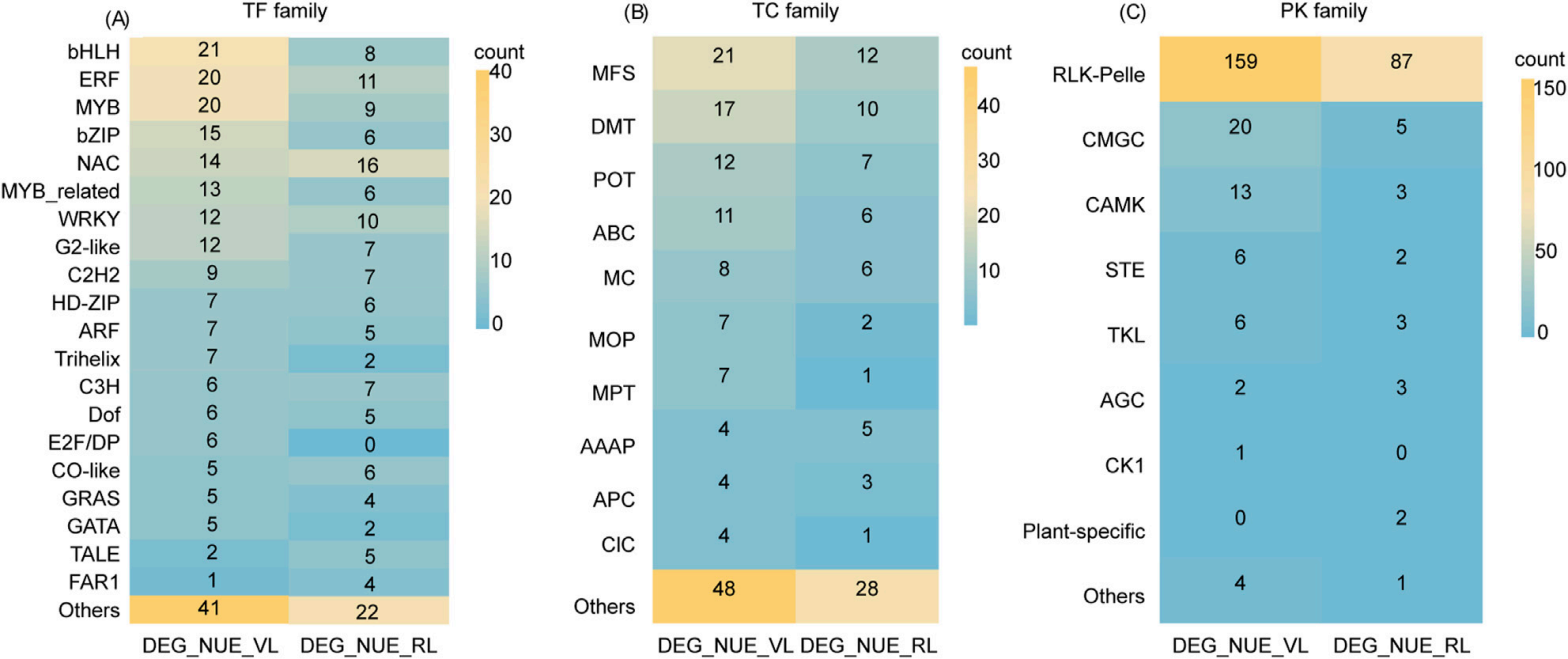
A transcription factor (TF) family analysis **(A)**, transporter (TC) family analysis **(B)**, and protein kinase (PK) family analysis **(C)** were conducted for DEG_NUE_VL and DEG_NUE_RL. DEG_NUE_VL and DEG_NUE_RL represent the differentially expressed genes (DEGs) that show significant correlations with nitrogen use efficiency (NUE-related) in the vegetative and reproductive stages, respectively.

We identified a total of 234 and 148 TFs in DEG_NUE_VL and DEG_NUE_RL, respectively, using PlantTFDB (Version 5.0) (Supplementary Table S6). The 234 TFs in DEG_NUE_VL were classified into 44 families, comprising 18 major classes (with at least four genes) with a total of 190 genes and 26 minor classes (fewer than four genes) with a total of 44 genes. Similarly, the 148 TFs in DEG_NUE_RL were categorized into 34 classes, including 17 major classes with a total of 122 genes and 17 minor classes with a total of 26 genes. Thirty-three major families were common in both DEG_NUE_VL and DEG_NUE_RL including bHLH, ERF, MYB, bZIP, NAC, MYB_related WRKY, and G2-like (Figure 7A). The analysis of expression differences revealed that 1.28%–70.09% of the TFs in DEG_NUE_VL exhibited significant regulation across the six DEG groups in the vegetative stage, with 0.42%–52.56% showing downregulation (Supplementary Table S6). Similarly, 2.02%–85.14% of TFs in DEG_NUE_RL were found in the six DEG groups in the reproductive stage, with 1.35%–39.19% showing significant downregulation.

A Venn analysis was performed to compare the identified TFs of NUE-related DEGs to NUE_grain_-related TFs reported in previous studies (Kumari et al., 2021; Sharma et al., 2023). The analysis revealed that six TFs in DEG_NUE_VL and two TFs in DEG_NUE_RL had been identified before (Supplementary Table S6). This suggests that our analysis provides numerous TFs candidates potentially involved in NUE under drought stress.

### 3.9 Transporters in NUE-related DEGs

Of the genes in DEG_NUE_VL and DEG_NUE_RL, 143 and 81 were identified as transporters, with 42 and 33 families in the Rice Transporters Database, respectively (Supplementary Table S7). The 42 families for DEG_NUE_VL included 10 major classes (with at least four genes) and 32 minor classes (fewer than four genes), while the 33 families for DEG_NUE_RL comprised 6 major and 27 minor classes. Notably, the major classes MFS, DMT, POT, ABC, MC, and AAAP were common to both sets of NUE-related DEGs (Figure 7B). The analysis of expression differences revealed that 0.70%–72.73% of the transporters from DEG_NUE_VL showed significant regulations across the six DEG groups in the vegetative stage (Supplementary Table S7). For DEG_NUE_RL, none of the transporters appeared in WM3_vs._WS3, while the other five DEG groups in the reproductive stage contained 1.23%–87.65% of transporters showing significant regulations.

Our analysis identified four transporters from DEG_NUE_VL and five transporters from DEG_NUE_RL that were reported as NUE_grain_-related transporters before (Kumari et al., 2021; Sharma et al., 2023) (Supplementary Table S7). This suggests that our findings highlight several transporters as potential candidate genes for enhancing NUE under drought stress conditions.

### 3.10 PKs in NUE-related DEGs

In the iTak database, 211 genes from DEG_NUE_VL and 106 genes from DEG_NUE_RL were identified as PKs. These PKs were classified into eight groups for both DEG_NUE_VL and DEG_NUE_RL (Figure 7C). Notably, the RLK-Pelle group had the highest number of PKs, accounting for 75.36% of DEG_NUE_VL and 82.08% of DEG_NUE_RL (Supplementary Table S8). The analysis of expression differences revealed that none of the PKs from DEG_NUE_VL appeared in WF2_vs._WM2 and WM1_vs._WS1, while 20.38%–71.09% were present in the other four DEG groups in the vegetative stage (Supplementary Table S8). Among these, 15 PKs exhibited significant regulation in all four of these groups, with 12 of them being downregulated. For DEG_NUE_RL, no PKs showed significant regulation in WF3_vs._WM3 (Supplementary Table S8). However, 0.94%–91.51% of PKs from DEG_NUE_RL were significantly regulated in the other five DEG groups in the reproductive stage.

Three PKs from DEG_NUE_VL and one PK from DEG_NUE_RL were identified as NUE_grain_-related PKs in previous studies (Kumari et al., 2021; Sharma et al., 2023) (Supplementary Table S8). This suggests that our analysis identifies several candidate PKs that could potentially contribute to enhancing NUE under drought stress.

## 4 Discussion

### 4.1 Rice growth under drought stress

Soil water availability plays a crucial role in influencing nutrient dynamics and use efficiency through various mechanisms, such as nutrient transformation, nutrient loss mechanisms, and microbial activity (Schimel, 2018; Ullah et al., 2019). In our study, we observed that soil NO_3_^−^-N concentrations were lower in normal irrigation treatments compared to drought stress treatments throughout the entire growth stage, while soil NH_4_^+^-N concentrations were higher in the normal irrigation treatments, except in the jointing stage (Figure 1). This trend aligns with findings from previous research (Reddy et al., 1984). The soil water contents in mild and severe drought stress conditions were maintained at 80% and 60% of the field capacity, respectively, which have been reported to partially inhibit and provide suitable moisture for soil nitrification (Zhang et al., 2002). Soil denitrification typically occurs when the soil water content exceeds 60% of field capacity. The optimal matric suction for N mineralization falls within the range of 1/3 to 0.1 bar, which overlaps with the soil water potential range observed in the drought stress treatments in our study (Stanford and Epstein, 1974). Furthermore, compared to the normal irrigation treatment, the available N (inorganic N) contents in mild and severe drought stress treatments were higher in the vegetative stage. However, in the reproductive stage, there were no significant differences in available N among the three treatments. This observation might be attributable to inhibited microbial activities caused by long-term drought stress in the mild and severe drought stress treatments, potentially suppressing the generation of soil inorganic N.

To cope with drought stress, plants self-regulate of their physiological and morphological traits. Drought stress typically results in reduced biomass production and yield (Farooq et al., 2009; Pandey and Shukla, 2015). In our study, we observed that the fresh weight and dry weight of aboveground rice under drought stress were consistently lower than those in the normal irrigation treatment throughout the entire growth period (Figures 2B, C), reducing the yields by 23.93%–25.40% (Table 1). These adverse environmental effects lead to growth retardation and subsequent yield losses. This growth retardation is primarily manifested through reduced whole-plant biomass accumulation, to conserve adequate energy reserves (Zhang et al., 2020). Additionally, drought stress imposes limitations on nutrient uptake. Drought stress treatments were associated with lower N accumulation compared to the normal irrigation treatment across the entire growth period; this was particularly evident in the jointing and maturing stages (Figure 2E). N uptake and utilization are energy-dependent processes (Grossman and Takahashi, 2001). Therefore, reduced N accumulation in plants under drought stress can lead to decreased energy consumption, which ultimately benefits plants in adapting to adverse environmental conditions. Despite the lower N accumulation observed in the drought stress treatments, the aboveground N concentration was higher compared to the normal irrigation treatment (Figure 2D). Aboveground N accumulation is simultaneously influenced by both aboveground N concentration and aboveground dry weight. This suggests that the lower aboveground N accumulation under drought stress may be attributable to the reduced aboveground dry weight. This finding is consistent with previous studies (Somaweera et al., 2016) emphasizing the critical role of biomass production in facilitating N accumulation. The drought stress in our study was due to a lack of irrigation. If rice is grown under drought stress caused by high temperature or low humidity, rice will suffer drought and high temperature stresses simultaneously, thus the adverse effects on rice growth and NUE will be more serious (Lawas et al., 2018; Wang et al., 2024). This may be a direction for future research because of global warming.

### 4.2 Rice NUE under drought stress

NUE reflects the efficiency of the plant in converting unit N into biomass. Consistent with previous research (Somaweera et al., 2016), we found that rice subjected to drought stress treatments exhibited significantly lower NUE compared to rice under normal irrigation treatment (Figure 2F). This indicates that drought stress restricts the efficiency of rice in converting N into dry matter, suggesting that rice plants prioritize the stress response over biomass production under such conditions. There may be increased amounts of stress-related nitrogenous compounds and their precursors, such as proline, betaine, glutathione, glutamate, and glycine, to serve as osmotica to help plants defend against drought (Ashraf and Foolad, 2007; Wingler et al., 2000; Cai et al., 2008; Li et al., 2010). Drought stress can inhibit photosynthesis through stomatal limitation and enhance photorespiration to relieve increased photoinhibition (Zhong et al., 2017). The inhibition of photosynthesis leads to a reduction of biomass (Du et al., 2020), and the enhancement of photorespiration can promote the photorespiratory N cycle, thus storing N in the forms of amino acids (Igarashi et al., 2006). This increased N storing and decreased biomass may lead to a reduction of NUE. Interestingly, despite the increase in available soil N under drought stress treatments, the rice plants still accumulated less N and exhibited lower NUE. This suggests a complex relationship between available soil N and rice NUE, emphasizing the significant impact of water conditions on NUE in rice. Future experimental investigations are needed to validate the impact of drought on rice NUE found in our study.

### 4.3 Transcriptional regulation under drought stress

Transcriptomics has been widely used to study the complex mechanisms in the response of plants to drought stress at the molecular level. Various studies have explored crop responses to only one degree of drought stress, including rice (Lv et al., 2019), watermelon (Yang et al., 2016), and tobacco (Yin et al., 2015), and the response to different levels of stress may be ignored. Additionally, previous transcriptome studies in plants under drought stress often focused on individual stage, such as the seedling stages (Park and Jeong, 2023; Zhang et al., 2017), and the reproductive stage (Kaur et al., 2023). In our study, we observed that the number of DEGs was consistently lower in the mild stress treatment compared to the severe stress treatment in various stages, except in the maturing stage (Figure 3A). A previous study focusing on the tillering stage found that the DEG number under drought stress compared to the control treatment was close to 4,000 (Zhou et al., 2022), which is consistent with the DEG number under severe drought stress compared to normal treatment in the tillering stage in our study. However, the number of drought-responsive genes in potting conditions (Figure 3A) was less than that in nutrient solutions (Park and Jeong, 2023; Zhang et al., 2017), which may indicate various responses to drought stress in different culture conditions. Compared with more than 10,000 drought-responsive genes in rice panicles at the reproductive stage (Kaur et al., 2023), the present study identified fewer drought-responsive genes in rice leaves at the reproductive stage, which may be due to different tissues of measurement. Additionally, only 36 genes exhibited significant expression differences across all stages (Supplementary Figure S3B). These findings suggest that rice undergoes distinct transcriptome regulation in response to different stages of growth and varying levels of stress.

### 4.4 Molecular mechanism for NUE under drought stress

WGCNA can be employed to connect genes to NUE phenotype to delve into the gene regulatory network of NUE (Langfelder and Horvath, 2008; Shanks et al., 2022). In our study, we identified 4,424 and 2,452 genes as NUE-related DEGs in the vegetative and reproductive stages, respectively. These genes potentially constitute crucial molecular mechanisms relevant to NUE under drought stress. Notably, glyoxylate and dicarboxylate metabolism and carbon fixation in photosynthetic organisms were the two enriched pathways shared by the two NUE-related DEGs (Figure 5D). These pathways exhibited significant enrichment specifically in NUE-related DEGs in the reproductive stage. The essential roles of carbon fixation in photosynthetic organisms and glyoxylate and dicarboxylate metabolism in responding to drought stress have been extensively validated at both transcriptome and physiological levels. Calvin cycle, the primary mechanism of carbon fixation in photosynthetic organisms in rice, can be disrupted by drought in multiple ways, which involve reduced CO_2_ concentration, decreased enzyme activities, and impeded transport of photosynthetic products (Oyebamiji et al., 2022; Qiao et al., 2024). Photorespiration, a pivotal module within the glyoxylate and dicarboxylate metabolism pathway, plays an important role in response to drought, which is involved in the generation of H_2_O_2_, the readjustment of redox homeostasis, and the scavenging of reactive oxygen species (Voss et al., 2013). An integrative analysis of physiology, transcriptome, and metabolism revealed that the decreased photosynthesis in a drought-tolerant cultivar caused by drought is not due to stomatal limitation, it is related to enhanced photorespiration and impaired photosynthetic apparatus (carbon fixation and photosynthesis-antenna proteins) (Zhou et al., 2022). These pathways are integral processes of carbon metabolism. Moreover, the interplay between carbon and N metabolism in plants has been a subject of investigation by numerous researchers, especially the collaborative response under drought stress. Photosynthesis requires a significant amount of N to construct the necessary protein systems, emphasizing the necessity of balancing carbon gain with the synthesis of organic N compounds (Evans and Clarke, 2019). Drought stress disturbs the balance of carbon and N metabolism (Cui et al., 2019). Soil drying can increase N metabolism which can improve photosynthetic acclimation to short-term water stress in rice (Zhong et al., 2018). N allocation in the bioenergetics and carboxylation system involves in the trade-off between the water stress acclimation and photosynthetic N use efficiency in rice (Zhong et al., 2019b). Our results suggest that carbon fixation in photosynthetic organisms, as well as glyoxylate and dicarboxylate metabolism, play important roles in the response of rice NUE to water stress conditions.

Carbon fixation in photosynthetic organisms involves the conversion of inorganic carbon (carbon dioxide) into organic compounds. One of the primary mechanisms responsible for this process in plants is the Calvin–Benson cycle (Fuchs, 2011). In this cycle, the enzyme Rubisco catalyzes the fixation of carbon dioxide by RUBP. Notably, the enzyme Rubisco can also promote RUBP to combine with oxygen for photorespiration, a vital mechanism of glyoxylate and dicarboxylate metabolism. The direction of the catalytic reaction depends on the ratio of carbon dioxide to oxygen. The impact of drought stress on rice Rubisco activity varies depending on the stage and severity of the stress. In rice in the vegetative stage, Rubisco activity significantly decreased under 30% PEG treatment (Zhou et al., 2007), while it increased under conditions of 45% field capacity and experienced a temporary boost under soil water potentials ranging from −10 to −40 kPa (Perdomo et al., 2017; Wang et al., 2022b). Studies have shown that Rubisco activity is typically downregulated in the reproductive stage under drought stress (Ji et al., 2012). In our study, we observed that the expression of *OsRBCS2-5* was induced by drought stress in the vegetative stage but was inhibited in the reproductive stage (Figures 5E, F). This finding is consistent with the results reported by previous researchers (Ji et al., 2012; Perdomo et al., 2017). Interestingly, we found that these four genes were negatively correlated with NUE in the vegetative stage and positively correlated in the reproductive stage (Figures 5E, F). Rubisco serves as an important N reservoir, containing approximately 25% of the leaf N. Therefore, a higher Rubisco content implies more N storage, potentially resulting in lower NUE. This could explain the negative correlation in the vegetative stage. However, a literature review revealed that rice plants with increased Rubisco content, such as RBCS-overexpressing rice plants, showed enhanced N absorption during the ripening stage, ultimately leading to higher NUE and increased yield (Yoon et al., 2020). This indicates a positive correlation between Rubisco and NUE, which aligns with the results of our study at the reproductive stage. Overexpression of *OsRBCS2-5* in rice may have the possibility to enhance NUE under drought stress by promoting Rubisco activity.

RUBP regeneration is another critical step in the Calvin–Benson cycle, and glyceraldehyde-3-phosphate dehydrogenase (GAPDH) is one of the key enzymes involved in this process. GAPDH catalyzes the reduction of 1,3-bisphosphoglycerate to glyceraldehyde 3-phosphate. Previous studies have reported an increase in GAPA protein abundance in rice under PEG treatment (Chintakovid et al., 2017) while the protein abundance of GAPB can be regulated by saline conditions (Chang et al., 2015). This modulation may contribute to the observed increase in GAPDH activity under PEG treatment (Cheng et al., 2015). In our study, we observed that the expression patterns of *GAPA* and *GAPB* were similar to those of *OsRBCS2-5*. Specifically, the expression of *GAPA* and *GAPB* in the vegetative stage was consistent with the findings of previous research (Cheng et al., 2015). Our study also revealed that *GAPA* and *GAPB* play crucial roles in NUE under drought stress. Thus, further research is needed to elucidate the mechanisms underlying the activities of Rubisco and GAPDH in influencing NUE under drought. Future research can explore the response of rice NUE with knockout or overexpression of *GAPA, GAPB,* and both to drought.

Photorespiration stands as a pivotal module within the glyoxylate and dicarboxylate metabolism pathway, demanding energy while potentially resulting in carbon and N depletion (Peterhansel et al., 2013). This process relies on glycolate, transported from chloroplasts to peroxisomes, which originates from 2-phosphoglycolate (2-PG), a byproduct of the oxygenating action of Rubisco on RUBP. Glyoxylate is transformed into 3-phosphoglycerate (3-PGA) through various metabolic pathways involving glycine, serine, and others before contributing to RUBP regeneration (Dellero et al., 2016). Notably, our study revealed similar expression patterns in key enzyme genes, such as *OsPGLP1*, *GLO1*, *GLO4*, *GLO5*, *OsGDCH*, and *OsSHM1*, mirroring the expression of *OsRBCS2-5* (Figures 5E, F), supporting the notion that the photorespiration response varies in intensity with increasing stress levels (Abogadallah, 2011; Chen et al., 2018). Additionally, GS and GOGAT were observed to play significant roles in our investigation, with *OsGS2* and *Fd-GOGAT1* showing congruent expression patterns with key enzyme genes in photorespiration (Figures 5E, F). Given the substantial release of ammonia during glycine conversion in photorespiration (Keys et al., 1978), the upregulation of *OsGS2* and *Fd-GOGAT1* in the vegetative stage in our investigation appears to be crucial for assimilating excess ammonium and mitigating N loss (Kendall et al., 1986; Somerville and Ogren, 1980). Moreover, the expression patterns of photorespiration genes aligned with those of Rubisco and GAPDH activities in our study, suggesting heightened activities of Rubisco, GAPDH, and photorespiration during the vegetative stage, potentially diminishing during the reproductive stage. During the vegetative stage, increased Rubisco activity and improved RUBP regeneration may expedite the Calvin cycle, but they also provide a large number of substrates and catalytic enzymes for photorespiration, potentially leading to the accumulation of toxic substances such as 2-PG, glycolate, and glyoxylate. It becomes crucial to augment the ‘detoxification’ role of photorespiration to support the growth of photosynthetic organisms. Conversely, during the reproductive stage, a reduction in toxic substances occurs as a result of decreased Rubisco activity and restricted RUBP regeneration. Consequently, the ‘detoxification’ function of photorespiration diminishes during this phase. These NUE-related DEGs associated with photorespiration may have great potential in breeding drought-resistant rice with high NUE.

Interestingly, *OsCATB* exhibited a distinct expression pattern compared to the aforementioned trend (Figures 5E, F). Catalase (CAT) enzymes are responsible for eliminating hydrogen peroxide, a significant reactive oxygen species. Approximately 70% of the total hydrogen peroxide in C3 plants is generated from photorespiration (Noctor et al., 2002). However, the expression profile of *OsCATB* did not align with those of other genes involved in photorespiration. This discrepancy suggests that the role of *OsCATB* in removing reactive oxygen species may not be related to drought (Joo et al., 2014).

A comprehensive empirical analysis revealed that high N levels under drought stress conditions can mitigate or even stimulate the biochemical processes of photosynthesis, such as Rubisco and GADPH activities, while preserving the photorespiration function in ammonia assimilation (Zhong et al., 2019a). In our investigation, drought stress was found to enhance soil inorganic N levels during the vegetative stage. This elevated soil inorganic N content could induce a high-N condition for rice plants experiencing drought stress, resulting in heightened Rubisco activity, increased GADPH activity, and improved photorespiration function in ammonia assimilation. Consequently, the increased expression of genes associated with Rubisco, GADPH, and GS/GOGAT during the vegetative stage may be attributable to the augmented soil inorganic N levels. This implies that the elevation in soil inorganic N induced by drought stress may also exert an influence on the molecular regulation of rice plants.

The two genes *GAPA* and *GAPB* were identified as hub genes in the vegetative stage. The *fructose-1,6-bisphosphate aldolase* (*OsAld-Y*) gene and seven other genes with unknown functions are the other eight hub genes in the vegetative stage. The knockout of *OsAld-Y* reduced chlorophyll accumulation and regulated sugar metabolism in rice (Zhang et al., 2016). In our study, drought stress induced the expression of *OsAld-Y* in the vegetative stage compared to normal irrigation (Supplementary Table S5). This is expected to improve chlorophyll accumulation, allowing more N to be stored in chlorophyll which may explain why the expression of *OsAld-Y* was negatively correlated with NUE in the vegetative stage. In the reproductive stage, the three genes *GLO4*, *GLO5*, and *OsGDCH* were identified as hub genes. The *aminotransferase* (*OsAMTR1*) gene and six other uncharacterized genes are the other seven hub genes in the reproductive stage. The expression of *OsAMTR1* showed significant induction under drought in a previous study (Kothari et al., 2016). Compared to normal irrigation, the expression of *OsAMTR1* was downregulated under mild drought stress and upregulated under severe drought stress in the reproductive stage. This indicates that *OsAMTR1* may have different functions in response to varying degrees of drought stress. These hub genes in the PPI network of NUE-related DEGs can be candidate genes for breeding drought-resistant rice with high NUE.

Furthermore, five genes associated with N metabolism were identified as NUE-related DEGs in both stages: *OsNRT1*, *OsAMT2.1*, *OsGS2*, *Fd-GOGAT1*, and *OsNADH-GOGAT2*. *OsNRT1* and *OsAMT2.1* are low-affinity nitrate and ammonium transporters, respectively (Lin et al., 2000; Suenaga et al., 2003). A previous study demonstrated that the upregulated expression of *OsNRT1,* activated by a MYB-like transcription factor, may be beneficial for improving crop NUE (Zhang et al., 2023). This is consistent with the positive correlation between NUE and the expression of *OsNRT1* during the reproductive stage in our investigation. The expression of *OsAMT2.1* was markedly upregulated in response to saline alkaline soil stress (Ma et al., 2022). In our study, the expression of *OsAMT2.1* was significantly downregulated under drought. This indicates that different abiotic stresses have various effects on the expression of *OsAMT2.1. OsGS2*, *Fd-GOGAT1*, and *OsNADH-GOGAT2* play important roles in ammonia assimilation. Some researchers found that the development of chloroplasts was impaired in *OsGS2* co-suppressed plants and *Fd-GOGAT1* mutants (Cai et al., 2010; Chen et al., 2016). The overexpression of *OsGS1;1* and *OsGS2* in transgenic rice was associated with enhanced photosynthetic and agronomic performance under drought imposed during the reproductive stage, ultimately leading to a higher yield (James et al., 2018). Together with our previous discussion, this indicates that *OsGS2* and *Fd-GOGAT1* are crucial in photorespiratory N metabolism. *OsNADH-GOGAT2* is involved in N utilization by affecting glutamine generation and N remobilization in senescent leaves (Yamaya and Kusano, 2014). This may be the reason that the expression of *OsNADH-GOGAT2* had a positive correlation with NUE in the reproductive stage in our investigation. Our study suggests that these five genes may have important functions in rice NUE in response to drought and the functions can be investigated by genetic manipulation of rice.

## 5 Conclusion

The current study delved into the impacts and underlying molecular mechanisms of drought on rice NUE. Drought stress was found to impede biomass and N accumulation, consequently leading to a reduction in rice grain yield. Specifically, compared to NUE under normal treatment, NUE under drought stress exhibited a significant decrease ranging from 2.18% to 31.67% across different growth stages. Totals of 4,424 and 2,452 genes were identified as NUE-related DEGs in the vegetative and reproductive stages, respectively. Notably, five genes associated with N metabolism were identified as NUE-related DEGs in both stages. Intriguingly, glyoxylate and dicarboxylate metabolism and carbon fixation in photosynthetic organisms were enriched (*p*-value <0.05) in NUE-related DEGs in both stages and exhibited significant enrichment (*p*-adjust <0.05) in NUE-related DEGs in the reproductive stage. The expression of some genes involved in these two pathways showed negative and positive correlations with NUE in the vegetative and reproductive stages, respectively. *GAPA* and *GAPB* emerged as hub genes among NUE-related DEGs in the vegetative stage, while *GLO4*, *GLO5*, and *OsGDCH* were identified as hub genes among NUE-related DEGs in the reproductive stage.

This study offers valuable information for understanding rice NUE under drought stress. Our results propose that five genes associated with N metabolism and several genes involved in the two significant enriched KEGG pathways (i.e., glyoxylate and dicarboxylate metabolism, as well as carbon fixation in photosynthetic organisms) in the two NUE-related DEGs are crucial in the regulation mechanism of rice NUE under drought. However, this study was carried out in a pot experiment that can not represent the environment in real-world agriculture, thus similar studies in field experiments should be conducted in the future. The response of rice NUE to the drought caused by high temperatures or no rainfall is also worth researching in the future due to global warming. The response across other rice varieties should also be considered. Additionally, further research is imperative to validate the functional roles of NUE-related DEGs regulating rice NUE under drought through genetic manipulation of rice, such as gene-editing strategies.

## Data Availability

The datasets presented in this study can be found in online repositories. The names of the repository/repositories and accession number(s) can be found in the article/Supplementary Material.
