# Supplementary material for: An integrated transcriptome and physiological analysis of nitrogen use efficiency in rice (Oryza sativa L. ssp. indica) under drought stress
Source: Front Genet. 2024 Nov 1;15:1483113. doi: 10.3389/fgene.2024.1483113 (PMC11564168; doi:10.3389/fgene.2024.1483113)
Supplement: Supplementary file 1 [file Presentation1.pdf]

Supplementary Figures

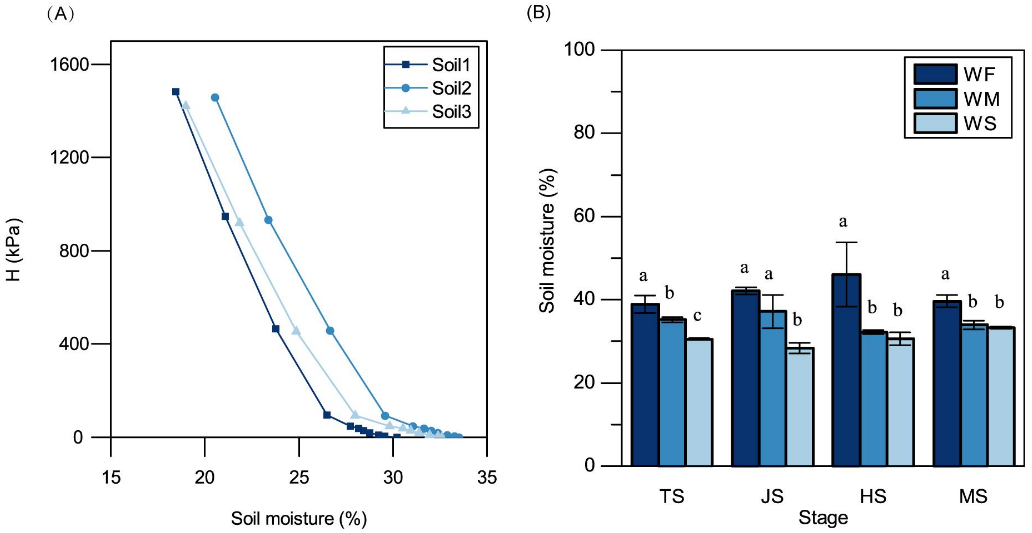

Supplementary Figure S1. The soil water retention curve **(A)** and the soil water moisture among three treatments at the four main growth stages **(B)**. Soil1, Soil2, and Soil3 were the three replicates for the experimental soil. WF, WM, and WS denote normal irrigation, mild drought stress, and severe drought stress, respectively, while TS, JS, HS, and MS represent the tillering, jointing, heading, and maturing stages. The data and error bars in the figure represent the average value and standard deviation of three repetitions, respectively. Different lowercase letters for the same growth stage denote significant differences at the 0.05 level by LSD test.

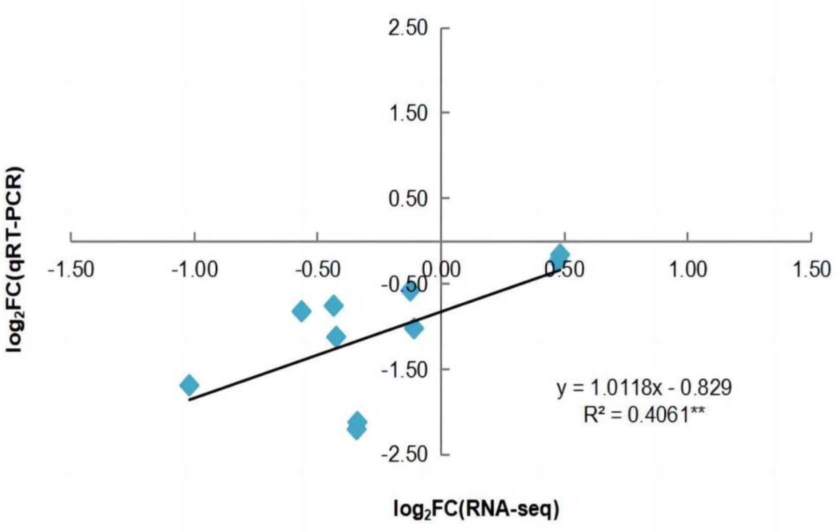

Supplementary Figure S2. qRT-PCR validation of DEGs in rice leaves. The *eEF1-α* gene was used as an internal control to normalize the expression data. The correlation coefficient between the log_2FC values obtained from RNA-seq and qRT-PCR results was calculated using the Pearson correlation coefficient.

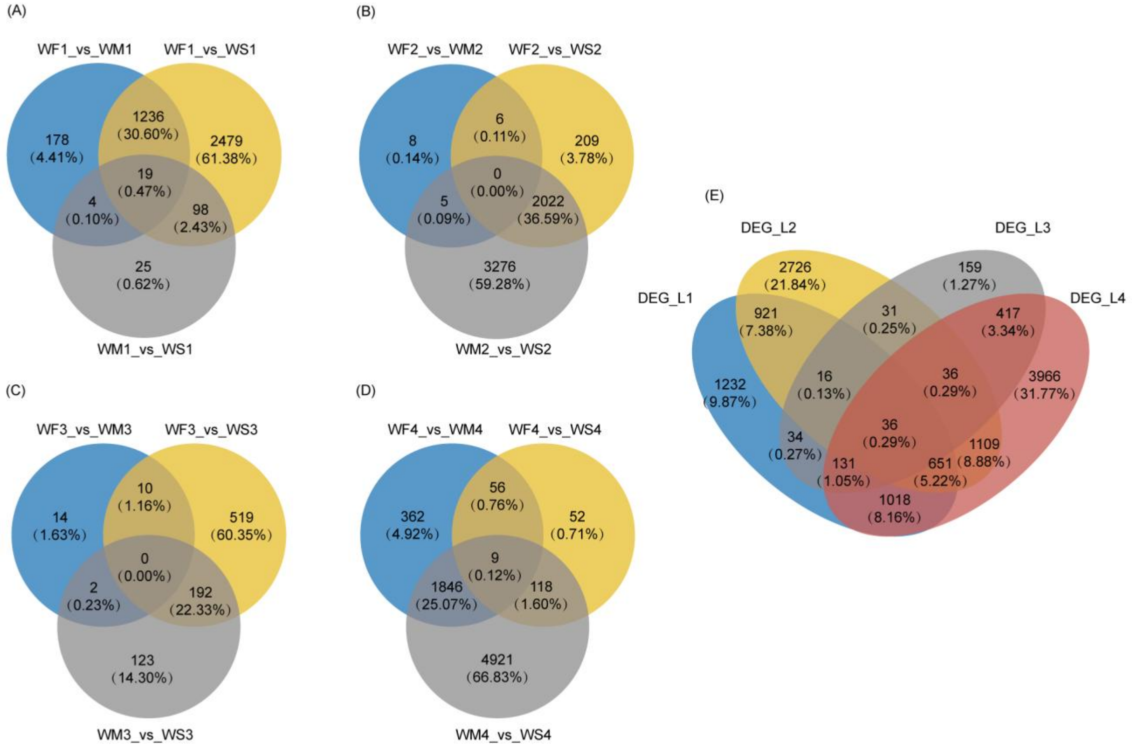

Supplementary Figure S3. Venn diagram of DEGs. **(A-D)** Venn diagram of differentially expressed gene (DEGs) among three difference groups at the tillering **(A)**, jointing **(B)**, heading **(C)**, and maturing stages **(D)**. **(E)** Venn diagram of DEGs among the four main growth stages. WF, WM, and WS represent normal irrigation, mild drought stress, and severe drought stress, respectively. 1, 2, 3, 4 denote the tillering, jointing, heading, and maturing stages, respectively. The comparisons WF_vs_WM and WF_vs_WS represent DEGs sets obtained by comparing WM and WS samples with the control WF samples, respectively. Similarly, WM_vs_WS denotes DEGs sets obtained by comparing WS samples with the control WM samples. DEG_L1, DEG_L2, DEG_L3, and DEG_L4 represent all the DEGs including WF_vs_WM, WF_vs_WS, and WM_vs_WS in the tillering, jointing, heading, and maturing stages, respectively.

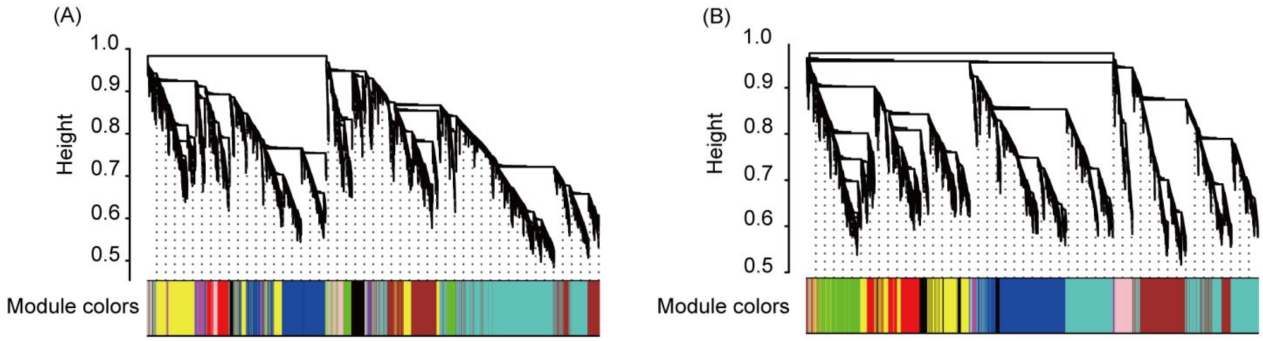

Supplementary Figure S4. Module identifications of WGCNA in the vegetative stage **(A)** and reproductive stage **(B)**. Genes are represented by the tips of the branches. The color band represents the module color in which the gene is grouped.
